# Comparison of adjuvant capecitabine plus oxaliplatin (CAPOX) versus S-1 after gastrectomy: a population-based cohort study using a nationwide claims database

**DOI:** 10.1038/s41598-023-44117-3

**Published:** 2023-10-11

**Authors:** Chi Hoon Maeng, Hoseob Kim, Mina Kim

**Affiliations:** 1 Division of Medical Oncology-Hematology, Department of Internal Medicine, Kyung Hee University Hospital, Kyung Hee University College of Medicine, (02447) 23 Kyungheedaero, Dongdaemun-gu, Seoul, South Korea; 2 Department of Data Science, Hanmi Pharmaceutical Co., Ltd, Seoul, South Korea

**Keywords:** Cancer, Gastric cancer

## Abstract

Although both capecitabine plus oxaliplatin (CAPOX) and S-1 are accepted as adjuvant chemotherapy following gastrectomy for gastric cancer, the better option between the two is still controversial. We conducted a retrospective nationwide cohort study using data from the National Health Insurance Service of Korea. We included patients who underwent gastrectomy for a primary diagnosis of gastric cancer between January 1, 2013, and December 31, 2018. The study compared the survival outcomes of patients who received postoperative chemotherapy based on S-1 (Arm S) vs. CAPOX (Arm C), as well as other relevant clinical variables such as comorbidity and completion of planned treatment. A total of 6602 patients were included in the analysis, with 4199 in Arm S and 2403 in Arm C. After propensity score matching, the final study population consisted of 2067 patients in each arm. Arm C showed statistically inferior 5-year overall survival (OS) and disease-free survival (DFS) rates compared to Arm S (84.0% vs. 90.0%; p < 0.0001; and 78.4% vs. 86.1%; p < 0.0001). Age (65 ≥ vs. < 65) and the incomplete planned treatment also had a significant negative effect on both OS and DFS. In the multivariable analysis, Arm C still showed worse OS (hazard ratio [HR], 1.609; 95% confidence intervals [CI], 1.339–1.934; p < 0.0001) and DFS (HR, 1.552; 95% CI 1.333–1.807; p < 0.0001) than Arm S. Both S-1 and CAPOX showed excellent efficacy, but this nationwide cohort study suggests that S-1 may be a better option in certain clinical situations.

## Introduction

Gastric cancer is a major type of cancer, representing a high disease burden both in incidence and mortality worldwide, including in Korea^1,2^. In Asia, the standard of care for locally advanced gastric cancer is still upfront gastrectomy with D2 lymph node dissection followed by adjuvant chemotherapy (AC), although neoadjuvant or perioperative chemotherapy before surgery is increasingly supported by evidence^3,4^. Both capecitabine plus oxaliplatin (CAPOX) and S-1 are accepted as standard ACs based on the Capecitabine and Oxaliplatin Adjuvant Study in Stomach Cancer (CLASSIC) and the Adjuvant Chemotherapy Trial of S-1 for Gastric Cancer (ACTS-GC) trials, respectively. CAPOX has the advantage of a shorter treatment period of 6 months compared to 1 year for S-1, but it requires intravenous injections of oxaliplatin every 3 weeks and has a higher incidence of adverse events of grade III or higher compared to S-1^5^. S-1 is an easily administered oral drug, but there are concerns that it may be less effective as a single agent than the platinum-based doublet, especially in patients with advanced-stage disease. This led to a study on the use of docetaxel in addition to S-1 in patients with stage III disease^6^. Furthermore, S-1 is mainly used in Asia and is not available in some regions, such as the United States. Accordingly, many researchers have published studies comparing these two treatments. However, these are heterogeneous and often show conflicting results. Many studies have reported similar efficacies of S-1 and CAPOX^5,7–11^. More specifically, on the other hand, subgroup analysis in some studies has shown that CAPOX was superior compared to S-1 in patients with high-risk or more advanced stages (e.g., stage IIIB or IIIC)^9,10^. Contrastingly, another study reported similar disease-free survival (DFS) and overall survival (OS) for S-1 and CAPOX in patients with stage III disease^5^. Another study found that CAPOX may be favorable for OS only in patients with stage II disease^8^. A recently published meta-analysis reported that the 5-year OS and DFS for stage II or stage III disease were not statistically different between the two treatments^12^. All these were retrospective studies. As a prospective phase III clinical trial comparing S-1 and CAPOX is unlikely to be conducted in the future, efforts to obtain convincing evidence for these two major types of AC should continue through analyses in as diverse a patient population as possible. Therefore, to investigated these two treatments in a larger patient group, we compared S-1 and CAPOX in a patient group registered in the National Health Insurance Service (NHIS) database of Korea.

## Results

### Study population

**Figure 1:**
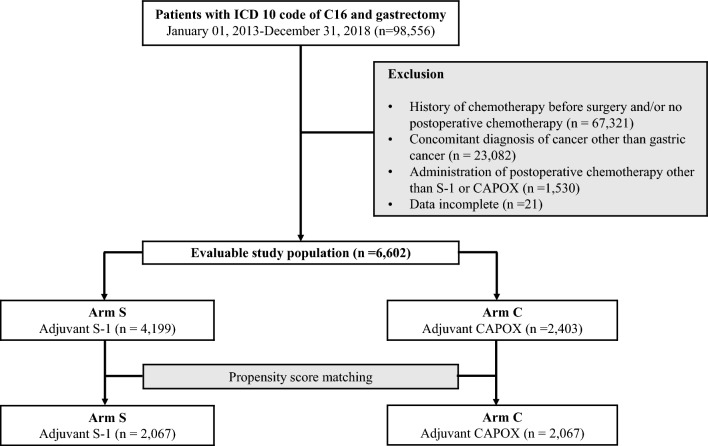
A flow diagram of study population selection. ICD, International Classification of Diseases; CAPOX, capecitabine plus oxaliplatin.

During the study period, 98,556 patients were identified as having undergone both gastric cancer and gastrectomy. Among this population, patients with a history of chemotherapy prescription before surgery and those who did not receive any chemotherapy after surgery (n = 67,321), who were diagnosed with cancers other than gastric cancer (n = 23,082), who received chemotherapy after surgery but were not treated with S-1 or CAPOX (n = 1530), and who had incomplete data (n = 21) were excluded. Consequently, 6602 patients were included in the analysis. Of these, 4199 patients received adjuvant S-1 (Arm S), and 2403 were treated with CAPOX (Arm C). After PSM, the final study population consisted of 2067 patients in each arm (Fig. 1). Table 1 summarizes the patients’ demographic characteristics. The median follow-up duration was 4.47 (range, 0.1–8.0) years.

**Table 1:** Baseline descriptive characteristics.

Characteristics		Before PSM								After PSM							
		Total		Arm S		Arm C		p-value	SMD	Total		Arm S		Arm C		p-value	SMD
		(N = 6602)		(N = 4109)		(N = 2403)				(N = 4134)		(N = 2067)		(N = 2067)			
		N	%	N	%	N	%			N	%	N	%	N	%		
Age (years)	< 30	25	0.4	13	0.3	12	0.5	< 0.0001	0.4473	8	0.2	4	0.2	4	0.2	1.000	0.0017
	≥ 30 and < 40	246	3.7	123	2.9	123	5.1			135	3.3	68	3.3	67	3.2		
	≥ 40 and < 50	915	13.9	500	11.9	415	17.3			669	16.2	335	16.2	334	16.2		
	≥ 50 and < 60	1825	27.6	1018	24.2	807	33.6			1389	33.6	694	33.6	695	33.6		
	≥ 60 and < 70	1859	28.2	1131	26.9	728	30.3			1339	32.4	669	32.4	670	32.4		
	≥ 70 and < 80	1487	22.5	1193	28.4	294	12.2			548	13.3	274	13.3	274	13.3		
	≥ 80	245	3.7	221	5.3	24	1.0			46	1.1	23	1.1	23	1.1		
	Median (range)	61 (17–93)		64 (17–93)		58 (22–86)				59 (29–86)		59 (29–86)		59 (29–86)			
Sex	Male	4468	67.7	2784	66.3	1684	70.1	0.0016	0.0812	2953	71.4	1477	71.5	1476	71.4	0.9725	0.0010
	Female	2134	32.3	1415	33.7	719	29.9			1181	28.6	590	28.5	591	28.6		
Income	Low income	1302	19.7	847	20.2	455	18.9	0.2845	0.0091	818	19.8	423	20.5	395	19.1	0.2089	0.0240
	High income	5191	78.6	3278	78.1	1913	79.6			3245	78.5	1603	77.6	1642	79.4		
	Missing	109	1.7	74	1.8	35	1.5			71	1.7	41	2.0	30	1.5		
CCI group	0–3	4405	66.7	2712	41.1	1693	25.6	< 0.0001		2919	70.6	1459	70.6	1460	70.6	0.9728	
	≥ 4	2197	33.3	1487	22.5	710	10.8			1215	29.4	608	29.4	607	29.4		
Completion of planned adjuvant chemotherapy	No	1798	27.2	1193	28.4	605	25.2	0.0045	0.0731	1014	24.5	485	23.5	529	25.6	0.1117	0.0481
	Yes	4804	72.8	3006	71.6	1798	74.8			3120	75.5	1582	76.5	1538	74.4		
Type of adjuvant chemotherapy	S-1	4199	63.6	4199	100.0		–			2067	50.0	2067	100.0	–	–		
	CAPOX	2403	36.4		–	2403	100.0			2067	50.0	–	–	2067	100.0		

PSM, propensity score matching; Arm S, S-1; Arm C, CAPOX; SMD, standardized mean difference; CCI, Charlson comorbidity index; CAPOX, capecitabine plus oxaliplatin.

### Survival outcomes

**Figure 2:**
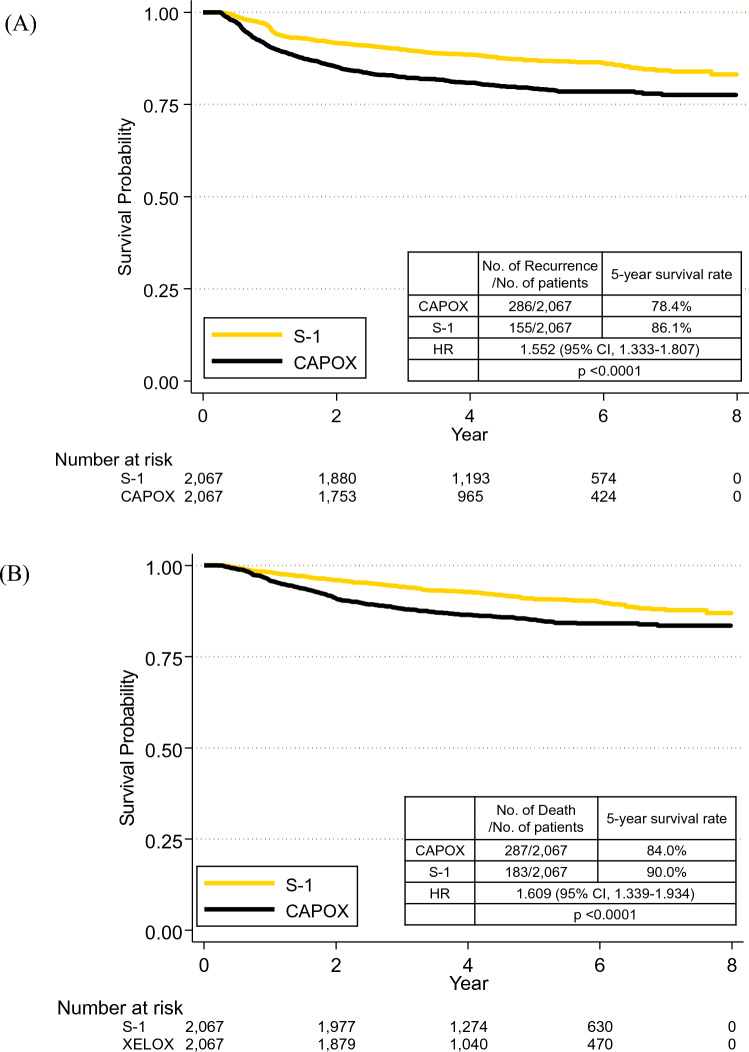
Survival outcomes based on the type of adjuvant chemotherapy. (**A**) Disease-free survival; (**B**) Overall survival. CAPOX, capecitabine plus oxaliplatin; HR, hazard ratio.

During the study period, disease recurrence occurred in 155 and 286 of the 2,067 patients in Arms S and C, respectively. Compared to Arm S, Arm C showed a shorter DFS (HR, 1.552; 95% CI 1.333–1.807; p < 0.0001). Five-year DFS rates were 86.1% in patients who received S-1 and 78.4% in those who received CAPOX (Fig. 2a). Deaths were confirmed in 183 and 287 of the 2,067 patients in Arm S and C, respectively. OS was also worse in Arm C than in Arm S (HR, 1.609; 95% CI 1.339–1.934; p < 0.0001). Five-year OS rates were 90.0% and 84.0% in arms C and S, respectively (Fig. 2b). Multivariable analysis revealed that younger age (< 65 vs. ≥ 65) and completion of planned AC were associated with better DFS and OS. Contrastingly, multiple comorbidities (≥ 4 vs. 0–3) did not affect the DFS or OS. After adjusting for clinically significant variables, CAPOX remained inferior to S-1 in terms of DFS and OS (Table 2).

**Table 2:** Univariable and multivariable analyses according to key variables.

Characteristics		DFS						OS					
		Univariable analysis			Multivariable analysis			Univariable analysis			Multivariable analysis		
		HR	95% CI	*p*-value	HR	95% CI	*p*-value	HR	95% CI	*p*-value	HR	95% CI	*p*-value
Age (years)	≥ 65 (vs. < 65)	1.773	1.524–2.064	< 0.0001	1.65	1.417–1.922	< 0.0001	2.57	2.147–3.076	< 0.0001	2.396	2.000–2.870	< 0.0001
Sex	Male (vs. female)	1.035	0.876–1.222	0.6884	1.06	0.897–1.253	0.4917	1.087	0.888–1.330	0.4188	1.119	0.914–1.371	0.2762
CCI group	≥ 4 (vs. 0–3)	1.172	0.724–1.896	0.5188	1.144	0.706–1.854	0.5853	1.547	0.925–2.587	0.0965	1.482	0.884–2.483	0.1355
Completion of planned adjuvant chemotherapy	Yes (vs. no)	0.343	0.295–0.399	< 0.0001	0.362	0.312–0.421	< 0.0001	0.303	0.253–0.363	< 0.0001	0.327	0.273–0.391	< 0.0001
Type of adjuvant chemotherapy	CAPOX (vs. S-1)	1.595	0.370–1.857	< 0.0001	1.552	1.333–1.807	< 0.0001	1.650	1.373–1.983	< 0.0001	1.609	1.339–1.934	< 0.0001

DFS, disease-free survival; OS, overall survival; HR, hazard ratio; CCI, Charlson comorbidity index; CAPOX, capecitabine plus oxaliplatin.

### Subgroup analysis

**Figure 3:**
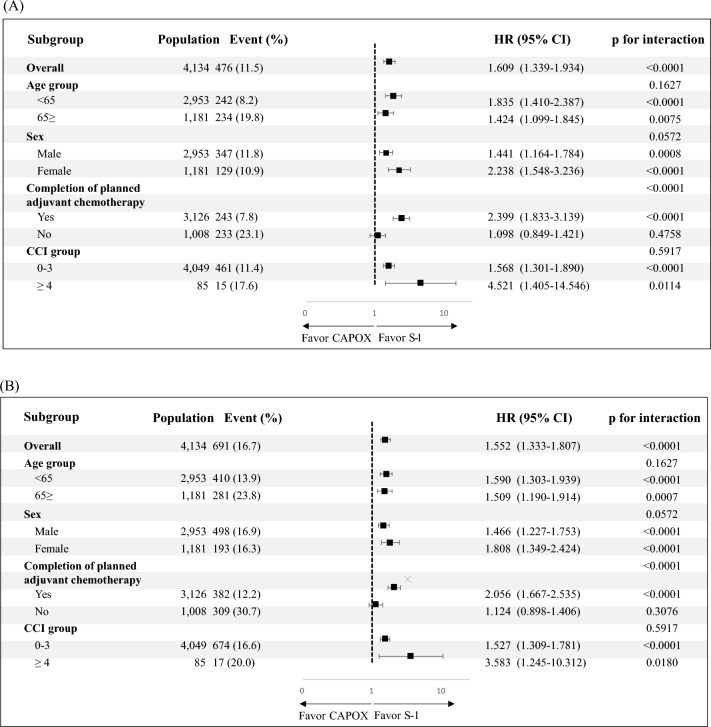
Forest plots of (**A**) disease-free survival and (**B**) overall survival according to patient subgroups. HR, hazard ratio; CCI, Charlson comorbidity index; CAPOX, capecitabine plus oxaliplatin.

In the forest plot, a consistent trend favored S-1 over CAPOX for DFS, regardless of the subgroup (Fig. 3a). A similar pattern was observed in the subgroup analysis for OS, favoring S-1 (Fig. 3b).

## Discussion

In this retrospective nationwide cohort study, we demonstrated a difference in efficacy between S-1 and CAPOX as AC following gastrectomy in patients with gastric cancer. To the best of our knowledge, this is the only study to date indicating that adjuvant S-1 is superior to CAPOX in patients with gastric cancer. Most published reports have shown that S-1 and CAPOX are comparable to each other^5,7–12^, or that better outcomes favor CAPOX, especially in patients with advanced-stage disease, in contrast to our study results^9,10^. In clinical practice, both of adjuvant S-1 and CAPOX are widely accepted and used. Some oncologists prefer CAPOX in cases of advanced-stage disease since subgroup analysis of the CLASSIC trial showed consistently favorable efficacy in patients with stages II, IIIA, and IIIB disease, whereas the effect of S-1 was maintained only in stage II disease in the subgroup analysis of the ACTS-GC trial^13,14^. Based on these results, the Korean practice guidelines for gastric cancer mention that CAPOX is the preferred choice for pathological stage II with regional lymph node metastasis or stage III disease^15^. Furthermore, based on several pivotal phase III studies, many clinicians now believe that fluoropyrimidine-based doublet chemotherapy has better efficacy than S-1 alone in these subgroups of patients^6,16^.

Why did Arm S showed better outcomes compared to Arm C in the present study? The discrepancy between our data and previous studies may be explained in some points of view. First, there is a possibility that the operational definition we used in this study could affect the capture of data on the status of disease recurrence, underestimating the actual number of recurrences. There is a subset patient who did not receive any type of palliative chemotherapy despite disease recurrence due to a number of clinical or non-clinical factors, including poor performance status, old age, or financial toxicity. As DFS was operationally defined as the period from the date of surgery to the start of first-line chemotherapy, patients whose cancer recurred but did not receive chemotherapy could not be included. Indeed, both the recurrence and mortality rates were much lower than those reported in previous studies. In the CLASSIC trial, 5-year DFS and OS rates after adjuvant CAPOX were 68% and 78%, respectively^17^. The corresponding rates were 65.4% and 71.7% after adjuvant S-1 in the ACTS-GC trial^18^. In real-world data, on the other hand, the recurrence rate after gastrectomy was found to be 19.7–20.5%, which was much lower than the results of clinical trials^19,20^. In our study, the 5-year recurrence rates were 13.9% and 21.6% in Arm S and C, respectively, which were slightly lower than or similar to those from real-world data. The difference between the datasets was considered reasonable, considering that the proportion of patients who did not receive palliative first-line treatment after recurrence was approximately 13% of all patients with recurrent or metastatic unresectable gastric cancer in Korea^21^. Nevertheless, because the type of prior AC itself cannot be considered as influencing the decision not to receive chemotherapy after recurrence, it can be assumed that the magnitude of underestimation of this portion might not be significantly different between the two groups. Additionally, despite the limitations of DFS estimation, the OS rates seem to be more robust in that the mortality information of the patients in this study was based on solid data from the NHIS rather than on an operational definition.

Second, it should be considered that in our data, patients with stages II and III diseases were mixed; therefore, analyzing the outcome by stage was not possible. Given the practice pattern of more frequent use of S-1 and CAPOX in patients with stages II and III disease, respectively, improved survival outcomes in Arm S are likely to be due to the effect of an earlier stage rather than the adjuvant S-1 itself. Unfortunately, detailed information on the disease stage was not available in the NHIS data, limiting the usefulness of these results. However, it is worth mentioning that our data suggested that both S-1 and CAPOX revealed high efficacy for patients in the adjuvant setting. An important thing is that, due to the limitation of unavailable information about the pathological stage, the results should be interpreted cautiously. If the distribution of stage II and stage III patients included in each arm was unbalanced, not only the type of AC, but also the imbalance in each arm was likely to affect the prognosis. Owing to this uncertainty, the following steps were taken to ensure data reliability: By performing PSM and including important variables available in the raw data, the imbalance of baseline demographic factors between the comparison groups was minimized. The superiority of S-1 over CAPOX was substantiated repeatedly, not only in the overall population but also in the subgroup analysis. These consistencies make the study results more convincing than a simple statistical coincidence. Moreover, we established strict criteria for selecting patients included in the analysis to minimize the intrinsic uncertainty in anonymized big data. For example, all patients whose diagnostic codes for gastric cancer (C16), and other cancer codes overlapped at least once were excluded, regardless of when the gastric cancer code was first generated. This is important because the current history of primary cancers other than stomach could make an accurate evaluation of AC for gastric cancer difficult. Apart from the type of chemotherapy, completion of chemotherapy and age were important prognostic factors, which is consistent with previous reports. However, the CCI score did not have a significant effect on survival outcomes. Collectively, these results suggest that efforts should be made to complete the planned course of chemotherapy as much as possible in adjuvant settings.

In conclusion, this study found that both adjuvants S-1 and CAPOX showed excellent efficacy in patients who underwent AC after gastrectomy. As revealed in the results, it can be seen that S-1 might be better than CAPOX, or at least S-1 may not be inferior to CAPOX even considering the aforementioned limitations of the data.

## Methods

### Study population and data source

For this retrospective nationwide cohort study, data were obtained from the NHIS of Korea. As the NHIS is a single-payer healthcare system, it covers the entire population of the Republic of Korea^22^ and provides comprehensive information on demographic data, healthcare utilization, pharmaceutical prescriptions, and death for each patient^23^. In the study population, we included patients who underwent gastrectomy [Q0251-Q0259, Q2533, Q2534, Q2536, Q2537, Q2552, Q2594, Q2598, QA536] with a primary diagnosis of gastric cancer certified by the International Classification of Diseases (ICD) 10th codes of C16.x from January 1, 2013, to December 31, 2018. Patients who underwent a gastrectomy before 2013 were excluded. We also excluded patients who (1) received chemotherapy before surgery and did not receive any chemotherapy after surgery; (2) had a diagnosis of other cancers, which was defined as patients with an ICD code other than gastric cancer (C16); and (3) received postoperative chemotherapy other than S-1 and CAPOX. This report complied with the Strengthening the Reporting of Observational Studies in Epidemiology (STROBE) reporting guidelines^24^.

Demographic factors included age, sex, income (0–29 vs. 30–100 percentile). Clinical variables such as comorbidity, type of AC (S-1 and CAPOX), completion of planned treatment, the time interval between surgery and the start date of adjuvant chemotherapy, DFS, and OS were also collected. Stages (II or III) of each patient were not available. The ICD-10 codes were utilized to define the comorbidities of the study population as follows: hypertension (I10–I13, I15), diabetes mellitus (E10–E14), dyslipidemia (E78), chronic kidney disease (N18), and stroke (I63–I64).

### Operational definition

Considering that the data were based on insurance claim data, the following operational definition was used to establish the predefined variables during data collection: AC was defined as the initiation of S-1 or CAPOX treatment within 3 months of surgery. If capecitabine and oxaliplatin were administered on the same day or at intervals of up to 1 week, it was defined that the adjuvant CAPOX was administered. To determine whether adjuvant S-1 was completed as planned, the reference period from the first administration start date to the last end date was estimated to be 336 days (42 days per cycle, eight cycles in total). If the administration of S-1 was finished between 30 days before and 60 days after the last day of the planned period, it was considered complete. In the case of CAPOX, if the number of oxaliplatin prescriptions was eight, the planned treatment was defined as complete, and if it was fewer than seven, it was considered incomplete. If the prescription of any chemotherapeutic agents was identified again after adjuvant S-1 or CAPOX was administered, it was defined as a recurrent case after surgery, and the patient received palliative first-line chemotherapy. Similarly, if another chemotherapy was prescribed during adjuvant S-1 or CAPOX, this was considered a case of recurrence during AC, and palliative first-line chemotherapy was initiated. DFS was defined as the period from the date of surgery to the start of first-line chemotherapy. Although we could not determine the actual date of radiologically or clinically confirmed recurrence, DFS was defined because palliative chemotherapy was initiated in cases of recurrence. OS was defined as the period from the date of surgery to the date of death. Data were not collected from patients who did not undergo chemotherapy, even if they relapsed, because they were unavailable.

### Ethical statement

This research was performed in accordance with the Declaration of Helsinki. The study protocol was reviewed and approved by the Institutional Review Board of Kyung Hee University Hospital (Approval Number: KHUH 2019-08-031), and the need for consent was waived. All personal information of the participants was anonymized and de-identified.

### Statistical analysis

Chi-square and t-tests were used to compare baseline characteristics, such as demographic characteristics and comorbidities, between patients who received adjuvant S-1 and CAPOX. To adjust for heterogeneity between the two groups, one-to-one propensity score matching (PSM) analysis was performed using standardized mean differences of 0.1. The variables used for PSM included age, sex, the region where patients received treatment, and the Charlson comorbidity index (CCI). OS and DFS were analyzed using the Kaplan–Meier method with the log-rank test. Hazard ratios (HRs) and 95% confidence interval (CI) values were estimated using the Cox proportional hazards model. All the statistical analyses were performed using SAS version 9.4 (SAS Institute, Cary, NC, USA). The level of statistical significance was set at p < 0.05.

### Supplementary Information

Supplementary Tables.

## Data Availability

Data are contained within the article and is available on request to the corresponding author, Chi Hoon Maeng.
