# Supplementary material for: Comparison of adjuvant capecitabine plus oxaliplatin (CAPOX) versus S-1 after gastrectomy: a population-based cohort study using a nationwide claims database
Source: Sci Rep. 2023 Oct 11;13:17154. doi: 10.1038/s41598-023-44117-3 (PMC10567838; doi:10.1038/s41598-023-44117-3)
Supplement: Supplementary file 1 — Supplementary Tables. [file 41598_2023_44117_MOESM1_ESM.docx]

# Supplementary Table 1. The first-line chemotherapy after recurrence

| **Characteristics** | |  | | **Before PSM** | |  | | **After PSM** | | | | | |
| --- | --- | --- | --- | --- | --- | --- | --- | --- | --- | --- | --- | --- | --- |
|  |  | **Total** | | **Arm S** | | **Arm C** | | **Total** | | **Arm S** | | **Arm C** | |
|  |  | **N** | **%** | **N** | **%** | **N** | **%** | **N** | **%** | **N** | **%** | **N** | **%** |
| *Single Agent chemotherapy* | | | | | | | | | | | | | |
| 5-fluorouracil |  | 6 | 0.9 | 1 | 0.3 | 5 | 1.5 | 3 | 0.7 | 0 | 0.0 | 3 | 1.0 |
| Capecitabine |  | 3 | 0.5 | 3 | 0.9 | 0 | 0.0 | 0 | 0.0 | 0 | 0.0 | 0 | 0.0 |
| Docetaxel |  | 2 | 0.3 | 1 | 0.3 | 1 | 0.3 | 1 | 0.2 | 0 | 0.0 | 1 | 0.3 |
| Doxifluridine |  | 132 | 19.9 | 100 | 29.9 | 32 | 9.8 | 83 | 18.8 | 53 | 34.2 | 30 | 10.5 |
| Irinotecan |  | 6 | 0.9 | 1 | 0.3 | 5 | 1.5 | 5 | 1.1 | 1 | 0.6 | 4 | 1.4 |
| Paclitaxel |  | 24 | 3.6 | 4 | 1.5 | 20 | 6.1 | 18 | 4.1 | 1 | 0.6 | 17 | 5.9 |
| S-1 |  | 52 | 7.9 | 28 | 8.4 | 24 | 7.3 | 29 | 6.6 | 10 | 6.5 | 19 | 6.6 |
| Tegafur |  | 33 | 5.0 | 31 | 9.3 | 2 | 0.6 | 11 | 2.5 | 10 | 6.5 | 1 | 0.3 |
| *Doublet Combination chemotherapy* | | | | | | | | | | | | | |
| DP |  | 7 | 1.1 | 2 | 0.6 | 5 | 1.5 | 5 | 1.1 | 1 | 0.6 | 4 | 1.4 |
| FOLFOX |  | 83 | 12.5 | 83 | 24.9 | 0 | 0.0 | 38 | 8.6 | 38 | 24.5 | 0 | 0.0 |
| FP |  | 11 | 1.7 | 8 | 2.4 | 3 | 0.9 | 7 | 1.6 | 4 | 2.6 | 3 | 1.0 |
| IP |  | 3 | 0.5 | 0 | 0.0 | 3 | 0.9 | 3 | 0.7 | 0 | 0.0 | 3 | 1.0 |
| SP |  | 16 | 2.4 | 0 | 0.0 | 16 | 4.9 | 15 | 3.4 | 0 | 0.0 | 15 | 5.2 |
| TP |  | 9 | 1.4 | 1 | 0.3 | 8 | 2.4 | 6 | 1.4 | 0 | 0.0 | 6 | 2.1 |
| CAPOX |  | 98 | 14.8 | 25 | 7.5 | 73 | 22.3 | 80 | 18.1 | 16 | 10.3 | 64 | 22.4 |
| XP |  | 2 | 0.3 | 2 | 0.6 | 0 | 0.0 | 0 | 0.0 | 0 | 0.0 | 0 | 0.0 |
| *Triplet Combination chemotherapy* | | | | | | | | | | | | | |
| FP-Trastuzumab | | 4 | 0.6 | 2 | 0.6 | 2 | 0.6 | 1 | 0.2 | 0 | 0.0 | 1 | 0.3 |
| XP-Trastuzumab | | 17 | 2.6 | 9 | 2.7 | 8 | 2.4 | 9 | 2.0 | 1 | 0.6 | 8 | 2.8 |
| Paclitaxel/ramucirumab | | 50 | 7.6 | 1 | 0.3 | 49 | 14.9 | 47 | 10.7 | 1 | 0.6 | 46 | 16.1 |
| *Others* | | | | | | | | | | | | | |
| Other platinum-base chemotherapy | | 5 | 0.8 | 3 | 0.9 | 2 | 0.6 | 3 | 0.7 | 2 | 1.3 | 1 | 0.3 |
| Not otherwise specified | | 99 | 15.0 | 29 | 8.7 | 70 | 21.3 | 77 | 17.5 | 17 | 11.0 | 60 | 21.0 |
| **Total** |  | 662 | 100.0 | 334 | 100.0 | 328 | 100.0 | 441 | 100.0 | 155 | 100.0 | 286 | 100.0 |

PSM, propensity score matching; Arm S, S-1; Arm C, CAPOX; DP, docetaxel/cisplatin; FOLFOX, oxaliplatin/leucovorin/5-fluorouracil;
FP, 5-fluorouracil/cisplatin; IP, irinotecan/5-fluorouracol; SP, S-1/cisplatin; TP, paclitaxel/cisplatin; CAPOX, capecitabine/oxaliplatin; XP, capecitabine/cisplatin

# Supplementary Table 2. The number of recurrence and death in each arm

| **Characteristics** | | **Before PSM** | | | | | | | | **After PSM** | | | | | | | |
| --- | --- | --- | --- | --- | --- | --- | --- | --- | --- | --- | --- | --- | --- | --- | --- | --- | --- |
|  |  | **Total** | | **Arm S** | | **Arm C** | | **p-value** | **SMD** | **Total** | | **Arm S** | | **Arm C** | | **p-value** | **SMD** |
|  |  | **N** | **%** | **N** | **%** | **N** | **%** |  |  | **N** | **%** | **N** | **%** | **N** | **%** |  |  |
| Recurrence | No | 5,958 | 90.2 | 3,883 | 92.5 | 2,075 | 86.4 | <0.0001 | 0.1842 | 3,693 | 89.3 | 1,912 | 92.5 | 1,781 | 86.2 | <0.0001 | 0.2050 |
|  | Yes | 662 | 10.0 | 334 | 8.0 | 328 | 13.6 |  |  | 441 | 10.7 | 155 | 7.5 | 286 | 13.8 |  |  |
| Survival | Alive | 5,703 | 86.4 | 3,633 | 86.5 | 2,070 | 86.1 | 0.6663 | 0.0110 | 3,664 | 88.6 | 1,884 | 91.1 | 1,780 | 86.1 | <0.0001 | 0.1465 |
|  | Dead | 899 | 13.6 | 566 | 13.5 | 333 | 13.9 |  |  | 470 | 11.4 | 183 | 8.9 | 287 | 13.9 |  |  |
| Total |  | 6,620 | 100.0 | 4,199 | 100.0 | 2,403 | 100.0 |  |  | 4,134 | 100.0 | 2,067 | 100.0 | 2,067 | 100.0 |  |  |
